# Consent, decisional capacity and guardianship in mental health research

**DOI:** 10.12688/wellcomeopenres.18003.2

**Published:** 2023-06-15

**Authors:** Juan Undurraga, Hanna Negussie, David Wendler

**Affiliations:** 1 Department of Neurology and Psychiatry, Faculty of Medicine, Clínica Alemana Universidad del Desarroll, Santiago, Chile; 2 Center for Innovative Drug Development and Therapeutic Trials for Africa, College of Health Sciences, Addis Ababa University, Addis Ababa, Ethiopia; 3 Department of Bioethics, NIH Clinical Center, Bethesda, MD, 20892, USA

**Keywords:** informed consent, clinical trials, decisional capacity, surrogates

## Abstract

**Background**: Research with adults who cannot give informed consent has important social value. However, enrolling adults who cannot consent in research raises significant ethical concerns.

**Methods**: To evaluate how researchers in low and middle-income countries (LMICs) can assess individuals’ decisional capacity, and the conditions under which it is appropriate, and the conditions under which it is not appropriate to include individuals who lack decisional capacity.

**Results**: In LMICs, where resources may be limited, implementing protections for adults with decisional incapacity can be especially challenging. Recognition of the ethical concerns, and awareness of the circumstances and available resources, offers the means to protect these vulnerable participants.

**Conclusions**: Researchers in low and middle-income countries should be aware of steps they can take to ensure appropriate protections for subjects with decisional impairments while conducting clinical trials on methods to improve their clinical care.

## Introduction

To give informed consent to enroll and continue to participate in clinical trials, individuals must possess a number of abilities to a sufficient degree. They must be able to understand the study in question sufficiently, including its purpose, risks, potential benefits, and alternatives, and they must be able to make and express a voluntary decision whether to participate based on this information and in light of their own preferences and values. Adults who are not able to do one or more of these things cannot consent for themselves. They should participate in clinical trials only when there is justification for their participation and provisions are in place to protect them.

Commentators use different terms to refer to individuals who cannot consent for themselves. Some refer to a lack of ‘decisional capacity’, others to ‘decisional competence’, and still others to ‘decisional authority’. Some use these terms interchangeably, while others consider decisional competence to involve a legal determination and decisional capacity to involve a clinical determination. Similarly, some commentators regard an inability to make a voluntary decision for any reason as rendering individuals unable to consent. Others consider inabilities which trace to factors internal to the patient (e.g. addiction), but not inabilities that trace to external factors (e.g. threats from others) to undermine the ability to consent. Given these terminological differences, it is critical for guidelines, policies and practice to clarify how the terms in question are being used. ^1^ For present purposes, we will use the term ‘decisional capacity’ to refer to individuals who satisfy all the conditions that are required to give informed consent and a lack of decision capacity to refer to individuals who do not satisfy one or more of these conditions.

To protect adults who lack decisional capacity, clinical trials should specify whether they may be enrolled, and whether they may continue to participate if they lose decisional capacity after they enroll. Clinical trials also need a process for assessing decisional capacity, including specification of who will be evaluated, how they will be evaluated, and who will evaluate them. Finally, if adults who lack decisional capacity may participate, additional safeguards should be incorporated into the study to protect them, including identification of someone (guardian or surrogate) who can give permission or consent on their behalf. The present manuscript presents two cases which illustrate these challenges as they arise in the context of research conducted in low and middle-income countries (LMICs) and considers ways to address them.

## Case 1. A first episode of psychosis clinic in Latin America

### Introduction

Psychotic disorders are associated with substantial morbidity and mortality, with a devastating effect on patients and their families ^1^. Because early intervention significantly improves response to treatment and long-term global functioning ^2, 3^, it has become a public health priority in first-episode psychosis (FEP) ^2, 4^. In LMICs, implementation of this approach faces significant hurdles, including a scarcity of resources, weak infrastructure, absence of mental health policies, few trained clinicians, and stigma ^5, 6^.

### Case study

In 2014, we established a cohort of FEP patients who were attending our clinic and were interested in participating in research. The goal of this research is to advance knowledge in this area and to obtain, through the research, resources that we can use to provide our patients with the best available clinical care (e.g. MRI, neuropsychological assessments). To date, we have conducted studies on metabolic syndrome and healthy lifestyle ^7^, neuroimaging ^8^, treatment resistance ^9^, social determinants of mental health ^10^, epidemiology ^11^, and public policies ^6^, among other topics. This case report describes the potential benefits and associated challenges of conducting research in the context of a clinical practice in Latin America that works with vulnerable populations who have difficulties with decisional capacity.

Chile has a mixed public-private health service, with approximately 80% of the population attending the public health system. The Chilean General Guarantee in Health Law, which took effect in 2005, ensures rapid access to standardized treatments and financial support for 80 prevalent conditions, including psychotic disorders ^4, 11^. Our program is based at the Instituto Psiquiátrico Dr. J. Horwitz, the largest psychiatric hospital in Santiago, the capital. With 22 inpatient beds and ambulatory services, the Early Intervention Program treats FEP patients between 16 and 30 years of age. It is a tertiary care center and patients are usually referred from emergency departments or by community health services. Our FEP patients typically present with severe symptoms that require inpatient care. During their hospitalization (mean= 32 days), patients are treated by a multidisciplinary team of psychiatrists, psychologists, social workers, occupational therapists, and nurses. In addition, families participate in periodic meetings and a structured psychoeducation program.

Participation in our follow-up research cohort involves additional sociodemographic and clinical evaluations, cognitive tests (MATRICS), a blood sample for DNA analysis, and a structural MRI, with a one-year ambulatory follow-up. To date, 132 of our FEP patients have consented to participate in the follow-up cohort, the largest first psychotic episode program in the country. Approximately 10%–15% of our patients have declined to participate. These patients continue with treatment as usual in our ambulatory clinic or in a community center following in-patient discharge.

A review of 12 studies of patients with schizophrenia, a mental illness that causes psychosis, found impaired decisional capacity in 10–52% of the sample, compared to 0–18% of controls ^12^. Impairments were more frequent in hospitalized patients, patients with greater cognitive impairment, and patients with more negative symptoms ^12^. These findings reveal that many people with schizophrenia have impaired decisional capacity. But, schizophrenia does not impair decisional capacity as a rule, highlighting the need to evaluate the capacity of individual patients. This need raises one of the primary ethical challenges we face: determining whether our patients are able to consent to participate in research.

While there is some debate, it is widely agreed that being able to consent to participate in clinical trials requires at least ^13^:

1. *Understanding* the study in question, including the purpose, risks, potential benefits, and alternatives;
2. *Appreciation* of how this information applies to one’s own condition and situation;
3. *Reasoning* based on this information and in light of one’s own values; and
4. *Expressing* a voluntary choice whether to participate.

Some patients clearly lack decisional capacity (e.g. unconscious patients, patients with severe thought disorders, patients in the advanced stages of dementia). In other cases, whether a patient has decisional capacity may be less clear. In these cases, we are morally committed to imposing minimal constraints on individual choice ^14^. In particular, it is important not to deny the right of patients who have decisional capacity to make their own decisions ^14^. On the other hand, it is critical to protect patients who lack decisional capacity. Therefore, we enroll adult patients in our research cohort only if they are able to give their own informed consent. In addition, we enroll 16 and 17-year olds who are able to give informed assent. This is consistent with Chilean law, which specifies that individuals under age 18 must provide assent.

Decisional capacity is “task specific”, meaning that it requires individuals to have the capacity to consent (or assent) to participate in a particular study. Individuals may have capacity with respect to some studies, but not others ^15^. In addition, decisional capacity can vary within a single individual over time. Recognizing this, we do not ask for consent to participate in our research when patients are admitted. Instead, we wait until the patient has been stabilized, and their decisional capacity is at its best, typically a few days before discharge.

At that point, the treating physician evaluates whether the patient meets the eligibility criteria and has decisional capacity. If so, she explains the study to the patient and asks for informed consent, or informed assent if the patient is 16 or 17-years old. The existence of a therapeutic relationship with the treating physician allows the patient and their caregivers to ask questions openly in a protected and trustful environment. We believe this approach permits a more comprehensive clinical and decisional capacity evaluation.

One of the most widely used instruments to help clinicians assess decisional capacity for research participation is the MacCAT-CR ^13^. It is based on the four abilities mentioned previously: comprehension, appreciation, reasoning, and expressing a voluntary choice. However, critics argue that the MacCAT-CR neglects other critical factors, especially the subject’s emotions, values, and the extent to which their choice accords with their values (i.e. authenticity) ^14^. Emotions, in particular, are an essential issue to consider in our patients, given evidence that some depressed patients have impaired capacity to weigh risks and potential benefits, even though they score well on the MacCAT-CR ^14, 16^. Based on these worries, we decided not to use standardized instruments, but to rely on the clinician who has been working with the patient to assess their understanding and capacity to consent to our research.

A second challenge faced by our team is the risk of coercion. Patients are invited to participate in the research cohort by their treating physicians. In addition, they are offered resources such as a cerebral MRI or cognitive evaluation that, otherwise, would not be available to them. This raises the worry that some of our patients might feel that they need to agree to participate in our research in order to receive care and access these extra resources.

To minimize this risk, we use an informed consent form and process that has been approved by the local Research Ethics Committee. This form tells patients and their families or caregivers that the clinicians have a dual role as treating clinician and researcher. The consent form and process also emphasize the voluntary nature of participation and individuals’ right to refuse to enroll or withdraw from the research at any time. Patients are also assured that their decision whether to enroll or refuse participation will not affect the therapeutic relationship or other benefits to which they are entitled according to the Chilean General Guarantee in Health Law. The fact that 10-15% of our patients decline to enroll suggests they do not feel that they must participate. Finally, our investigators do not receive any financial incentives for enrolling patients in research. We believe this minimizes the chances that they will pressure patients to participate in the research, although academic or personal incentives may still influence them.

### Reflections

Decisional capacity is heterogeneous within and across populations, and can vary within the same individual over time. In particular, there is evidence that people with mental health disorders have different degrees of decisional capacity. Patients should thus not be deemed to lack decisional capacity based on a diagnosis alone. As a research community, we should try to balance protection and non-discrimination by protecting a person’s decisional capacity and assessing it relative to a specific decision and context. We also aim to reduce behaviors that might undermine voluntariness, such as coercion.

Another critical issue is the ability of the researcher to conduct an accurate assessment of patients' decisional capacity. One way to improve this capacity is to use standardized assessment tools, but they have been criticized as insufficient. There is therefore need a to develop universally accepted procedures, especially for patients with mental health conditions. Another way to improve the researcher’s assessment ability is to rely on task-shifting. Our group does this by bringing complex cases to senior members or mental health specialists to obtain their input and to resolve on a case-by-case basis. Lastly, assessment of decisional capacity should be considered a process and done at multiple time points, if possible.

## Case 2. Task Sharing for the Care of Severe mental disorders in a low-income country: a randomized, controlled non-inferiority trial (TaSCS Trial)

### Introduction

Mental disorders are among the leading non-communicable disorders in terms of health burden in Ethiopia ^17^ and many individuals with severe mental disorders (SMDs) suffer long-term illness and disability ^18^. However, it is estimated that less than 10% of persons with SMDs in Ethiopia ever receive evidence-based treatment ^19^. This large treatment gap for SMDs, common in low-income countries of sub-Saharan Africa, is attributed to centralized services, which are frequently not accessible to rural people, as well as a critical shortage of mental health providers ^20^. In Ethiopia, outside the capital city, Addis Ababa, specialist mental health services are limited largely to psychiatric nurse-led, hospital-based clinics located at the district or regional level. In order to scale-up mental health services, most of the care of persons with SMDs will, therefore, need to be task shared with primary health care (PHC) and other general health care workers ^21^, with support from specialist mental health services.

A systematic review conducted by the World Health Organization (WHO) identified packages of mental health interventions (detailed in the Mental Health Gap Project Intervention Guide: mhGAP-IG) that have demonstrated effectiveness for mental, neurological and substance use disorders and can be delivered in the primary care setting ^22^. Provision of these packages has been advocated as a means to narrow the high treatment gap for people with mental health conditions in low-income countries. The expectation is that sharing these packages between primary care providers and mental health care specialists will make them more affordable and accessible to persons with SMDs compared to existing centralized provision of mental health care.

This approach to mental health care is at the centre of the Ethiopian National Mental Health Strategy (National Mental Health Strategy 2012/13 – 2015/16). However, little was known about how different aspects of the care of persons with SMDs can be safely and effectively transferred to the PHC setting in a rural, low-income country such as Ethiopia. In addition, the effectiveness and safety of this approach had not been evaluated using a randomized controlled trial in a low-income country.

### Case study

The Task Sharing for the Care of Severe mental disorders in a low-income country (TaSCS) trial was a randomized, controlled noninferiority trial. The goal of the trial was to investigate whether mental health care for people with SMDs integrated within primary health care using task-sharing was noninferior to a less accessible, but more specialized existing model of psychiatric nurse-led care. The study was conducted in Meskan and Mareko districts, Gurage Zone, and Silti Zone, Southern Nations, Nationalities and People’s Region, Ethiopia.

SMDs can undermine individuals’ decisional capacity ^23^. As a result, the treatment packages that are being scaled up by the Ministry of Health will inevitably be delivered to people with SMDs who lack decisional capacity. For this reason, we included people with SMDs who had decisional capacity to consent to participate and also people with SMDs who lacked decisional capacity. This design permitted us to test the interventions, particularly in relation to safety, in the populations who would be receiving them in the clinical setting.

Persons with SMDs who lack decisional capacity are less able to protect their own interests. Thus, procedures were established to safeguard them from possible harms arising from their involvement in the study. Psychiatric nurses were trained by experienced psychiatrists to assess individuals’ decisional capacity with respect to the study using a semi-structured form. However, common practice in Ethiopia is to administer treatment without assessing whether the patient has decisional capacity. Given this background, there was concern that assessment of decisional capacity is unfamiliar to most mental health practitioners in Ethiopia, which may have limited the quality of the assessments during the study, despite extensive training.

Another concern was obtaining permission from an appropriate guardian to enroll patients who were found to lack decisional capacity. The legal concept of ‘guardian’ is not widely used in Ethiopia, hence, we decided to rely on the patient’s caregivers. Information about the trial was explained to the person with the SMD and one of three caregivers who were documented by project outreach workers during a home visit. Individuals were enrolled if the caregiver gave permission for research enrollment and the patient did not object to being enrolled.

Caregivers play a vital role in facilitating access to care for people with mental health conditions in Ethiopia and many low-income country settings ^24^. However, previous studies from Ethiopia have indicated that the treatment priorities of people with mental health conditions and caregivers sometimes diverge ^25, 26^. The relationship between a person with a mental health condition and caregiver may also be complicated by the fact that caregivers are sometimes a party to coercive practices that are employed in the treatment setting, including restraint or covert administration of medications ^27^.

When caregivers are involved in providing permission for persons who lack decisional capacity, care is taken to ensure the caregiver (usually a close family member) lives with the individual and knows their preferences. The team also ensures that the person who lacks decisional capacity does not object to the caregiver taking on this role.

Decisional capacity can vary over time. Hence, to maximize the possibility that persons with a SMD could consent for themselves, the study design called for a formal reassessment of decisional capacity at baseline, 12 months, and 18 months, as well as at any time the family or a health care professional indicated in the clinical assessment sheets that the person might have regained capacity. In practice, the formal reassessments took place only at the specified trial time-points due to a lack of communication between trial staff, family members and health workers regarding possible changes in participants’ mental health status.

Similarly, given the possibility that participants might lose decisional capacity after they enrolled in the trial, people who had capacity at baseline were asked to complete an advance directive to guide what should happen if they subsequently lost capacity. They were asked whether they were willing to stay in the study if their mental health deteriorated to the extent that they lost the capacity to consent, provided the caregiver who brought the person to screening and registered as a caregiver provided permission. With the permission of the participant, the contact details of the caregiver were recorded at baseline so that they could be contacted if the person lost capacity during the course of the study. 

Another concern was whether consent to participate in the research, which could be withdrawn at any time, was distinguished clearly from consent to receive treatment. This may have been exacerbated by the absence of legislation in Ethiopia to protect the autonomy of people with mental health conditions with respect to deciding whether to accept mental health treatment. However, as the trial intervention was based on out-patient mental health care, it was unlikely to increase participants' exposure to coercive care beyond the existing risk.

To assess the possibility that participants in the study might receive inferior care, the Data Safety and Monitoring Board regularly reviewed proxy outcomes for potential inferiority of care and reviewed an interim analysis at 12 months. This potential risk was also disclosed in the information sheet and discussed during the consent process, including the measures which were in place to minimize it. In addition, project psychiatric nurses conducted weekly supervision of the primary care workers delivering task-shared care for the first 3 months in the intervention arm and reviewed their follow up clinical sheets. 

### Reflections

It is important to include people with mental health conditions in clinical trials, especially when the trial offers them the potential for clinical benefit. At the same time, it is important to protect people with mental health conditions from being exposed to excessive risks. The first step in this process is to assess whether the individuals have the capacity to consent to participate. For this purpose, it is important to ensure the researchers are well trained to conduct capacity assessments.

Decisions about who should provide permission for people who are found to lack decisional capacity are context dependent, and should be made in light of any local or national regulations. In the absence of legal guardians, it is vital to ensure the right individual is chosen to provide permission. Because decisional capacity can vary over time, there should be on-going assessments; the assessment should not be a one-time event, but rather a continual event, seeing the study participants regularly to conduct multiple assessments as appropriate. Finally, having participants with decisional capacity complete an advance directive at the time of research enrollment gives them the opportunity to direct how they will be treated if they later lose decisional capacity.

## Discussion

Historical abuses led to an emphasis on informed consent as a critical safeguard for research participants ^28^. Most notably, the Nuremberg Code describes informed consent as “essential” to ethical clinical trials ^29^. This approach provides important protection for adults who cannot give informed consent. However, since the Nuremberg Code, it has been recognized that this approach also may represent overprotection. In particular, prohibiting all research with adults who are unable to consent poses a significant barrier to studies needed to improve treatments for conditions associated with cognitive impairment, such as Alzheimer disease. To address this concern, the Declaration of Helsinki permits research with adults who cannot give informed consent provided the researchers obtain “informed consent from the legally authorised representative.” ^30^ Similarly, the CIOMS guidelines state that adults who cannot consent may be enrolled when a “legally authorized representative of the person who is incapable of giving informed consent has given permission and this permission takes account of the participant’s previously formed preferences and values (if any).” ^31^

Over the past 20 years, there has been significant discussion regarding the ethics of conducting research with individuals who cannot give informed consent, and what specific safeguards are needed to protect them ^32– 35^. One proposal endorses 7 safeguards: 1) Approval by the IRB/REC; 2) Appropriate risk–benefit profile; 3) Assessment of participants’ capacity to consent; 4) Justification for enrolling adults who cannot consent; 5) Permission of an appropriate proxy decision maker; 6) Consistency with participants' preferences and values and 7) Respect for participant assent and dissent ^32^.

To date, implementation of these protections has focused on high-income countries. In particular, guidelines specify that trained clinicians should assess the decisional capacity of potential research participants. Similarly, as reflected in the Declaration of Helsinki and the CIOMS guidelines, it is frequently mandated that the proxy decision maker should be authorized by local or national legislation to enroll adults in research.

These conditions are frequently satisfied in high-income countries. For example, the Department of Bioethics at the US NIH Clinical Center offers a 24/7 consultation service to intramural researchers. This service uses dedicated bioethicists and a task specific approach to assess whether potential research participants have the capacity to consent. ^2^ When individuals are found to lack decisional capacity, researchers rely on NIH policy and US federal research regulations to identify a surrogate.

It is, of course, critical to protect individuals in LMICs who cannot consent as well. However, resource constraints can make it difficult or impossible to implement the specific measures called for by existing guidelines. For example, LMICs may not have enough trained clinicians to assess decisional capacity nor legislation specifying who can serve as a research surrogate.

It does not follow, however, that it is impossible to conduct ethical research with adults who cannot consent in LMICs. Instead, as illustrated by the two cases presented here, it is important to step back from the specific procedures mandated by existing guidelines, identify the critical protections that they are intended to realize, and consider how they can be realized in the setting in question.

When there is an absence of trained psychiatrists, researchers should consider who is in a position to engage with and assess potential participants without coercing them or exerting undue influence. For example, can nurses be trained to conduct capacity assessments? These assessments should be based on recognition of the fact that a diagnosis does not determine whether individuals can consent. Instead, it is critical to conduct task specific assessments to determine whether the individual has the capacity to consent to the study in question.

Decisional capacity can vary over time. Individuals who cannot consent at the time of admission to the in-patient setting may be able to consent for themselves after their condition has stabilized. Moreover, consent capacity should be monitored to assess whether individuals who consent to research enrollment lose capacity over time due to specific treatments or the progression of their condition.

In the absence of legislation specifying who can serve as a research proxy, it is important to identify someone who can make decisions based on the preferences and values of the individual, and also protect their interests. The CIOMS guidelines state that, “in situations where a legally authorized representative is not available to allow for timely enrolment, researchers may obtain the permission of a representative who is socially accepted but not formally recognized before the law.” ^3^ While this statement seems to refer to cases where a legally authorized representative is not available due to time constraints, it raises the possibility of relying on representatives who are socially accepted in places where there are no laws specifying who qualifies as a legally authorized representative. For example, in Ethiopia, ethics committees frequently approve the caregiver, typically a close family member, who lives with the individual and knows their preferences as the surrogate.

Finally, enrolling individuals who cannot consent does not mean ignoring their current preferences and values. They should be involved in the decision-making process to the extent of their abilities and interest. Their assent should be obtained to the extent possible and their dissent should be respected. In this regard, when a caregiver is serving as the surrogate in Ethiopia, the team ensures that the person who lacks decisional capacity does not object to the caregiver taking on this role.

## Summary

Research with adults who cannot give informed consent is important to identifying effective and safe interventions for the conditions that affect them. Enrolling adults who cannot give informed consent also raises significant ethical concerns, highlighting the need for safeguards to protect and respect adults who cannot give informed consent. In LMICs, where resources may be limited, implementing these protections may be especially challenging. The two case studies discussed here reveal that recognition of the ethical concerns and awareness of the circumstances and available resources can provide means to protect these vulnerable participants while conducting valuable research needed to help them.

## Data Availability

No data are associated with this article.

## Footnotes

^1^ Scott Y.H. Kim. Evaluation of Capacity to Consent to Treatment and Research. Oxford: Oxford University Press, 2009.

^2^ Matera-Vatnick M, Todman KW, Wakim PG, Sullivan HK, Squires C, Brintnall-Karabelas J, Doernberg SN, Danis M. Evaluating the Ability to Consent to Research: A Twenty-Year Track Record. Ethics Hum Res. 2022 Mar;44(2):2–17. doi: http://www.doi.org/10.1002/eahr.500119.

^3^ Commentary 16
