# Supplementary material for: Consent, decisional capacity and guardianship in mental health research
Source: Wellcome Open Res. 2023 Jun 15;7:183. Originally published 2022 Jul 7. [Version 2] doi: 10.12688/wellcomeopenres.18003.2 (PMC10318371; doi:10.12688/wellcomeopenres.18003.2)
Supplement: Peer review history

# Peer review: Reviewer response for version 2

**DOI:** 10.21956/wellcomeopenres.21673.r60355

**Published:** 2023-07-03

**Authors:** Rieke van der Graaf

**Affiliations:** 1 Julius Centre of Primary Care and Health Sciences, Department of Medical Humanities, University Medical Centre Utrecht, Utrecht, The Netherlands

The authors have adequately addressed my comments. I have no further comments to make.

Does the article adequately reference differing views and opinions?

Yes

Are all factual statements correct, and are statements and arguments made adequately supported by citations?

Partly

Is the Open Letter written in accessible language?

Yes

Where applicable, are recommendations and next steps explained clearly for others to follow?

Yes

Is the rationale for the Open Letter provided in sufficient detail?

Yes

Reviewer Expertise:

Bioethics, research ethics, global health ethics

I confirm that I have read this submission and believe that I have an appropriate level of expertise to confirm that it is of an acceptable scientific standard.

# Peer review: Reviewer response for version 1

**DOI:** 10.21956/wellcomeopenres.19962.r56610

**Published:** 2023-05-22

**Authors:** Rieke van der Graaf

**Affiliations:** 1 Julius Centre of Primary Care and Health Sciences, Department of Medical Humanities, University Medical Centre Utrecht, Utrecht, The Netherlands

I have read the manuscript with great interest and highly appreciate the effort to write a paper on the basis of two cases presented at the Global Forum for Bioethics in Research conference in 2021. Happy to see that the case presenters are also first and second author and have been supervised in the writing process. The manuscript presents two cases on decisional capacity in an LMIC context and reflects on these cases. The case analyses and discussions enrich the existing guidance and models for research with incapacitated persons.

 Although the manuscript is well-written and I think a welcome contribution to the literature, I think there are a few issues that need some more attention:

1. It is not always clear what makes these cases unique for LMICs. For example, as regards case 1, the authors write on page 4 that they decided to not use standardized instruments to assess decisional capacity, such as the MacCAT-CR because they also want to give room for the role of emotions. But that’s not typical for an LMIC context. Also the fact that they are enrolled by the treating physician is not unique for LMICs. In case 2, I think it is not specific for LMICs that decisional capacity is re-assessed during the study, or to ask for advance directives when decisional capacity is likely to be impaired over time. One way to address this comment might be to say that these issues occurred in these cases and how they were dealt with, but to say that there are no indications that these issues are unique to LMICs, which might take away the misunderstanding that research ethics standards have to be weakened in LMICs because of resource constraints etc. For example, see this paper that was the result of a GFBR meeting ^1^, where the authors concluded “In our case studies, these issues did not seem to raise special ethical scrutiny in low-resource settings”.
2. In case 1, enrolment by the treating physician is preferred because it is thought to provide a more protected and trustful environment. That the dependent relationship can have beneficial aspects is well-recognized in the bioethics literature. At the same time, extra protections will remain necessary. See for instance CIOMS guideline 9: “However, in some situations of dependency, it is preferable that the clinician provide the patient with information since he or she is most knowledgeable about the condition of the patient. However, to minimize the influence of the dependent relationship, several protective measures must be taken. Clinicians engaged in research must acknowledge and inform patients that they have a dual role as the treating clinician and researcher. They must emphasize the voluntary nature of participation and the right to refuse or withdraw from the research. They must also assure patients that their decision whether to enrol or refuse participation will not affect the therapeutic relationship or other benefits to which they are entitled. In cases where it is necessary for the treating clinician to explain the details of the study protocol, the research ethics committee must consider whether the informed consent document must be signed in the presence of a neutral third party.” I’m interested to know what additional protections were taken to avoid the negative aspects of the dependency relationship. Also for case 2, some further elaboration on what is done to mitigate concerns when the caregiver fulfils the role of the “legal guardian” in Ethiopia would be good.
3. In case 1, several aspects are described related to coercion. “Patients are invited to participate in the research cohort by their treating physicians. In addition, they are offered resources such as a cerebral MRI or cognitive evaluation that, otherwise, would not be available to them.” Strictly speaking, coercion is related to a different element of informed consent (voluntariness), whereas decisional capacity is related to reasoning/understanding. Perhaps somewhat broaden the scope of the case examples (not confined to only decisional capacity).
4. As regards case 2, on page 6, there is a suggestion that the CIOMS guidelines have too little attention for LMIC- based circumstances. The authors write “Similarly, as reflected in the Declaration of Helsinki and the CIOMS guidelines, it is frequently mandated that the proxy decision maker will be authorized by local or national legislation to enrol adults in research. However, resource constraints in LMICs frequently make it difficult or impossible to follow these approaches. LMICs may not have enough trained clinicians to assess the decisional capacity of potential participants nor legislation governing who can serve as a research surrogate or proxy.” I think the commentary to guideline 16 provides more nuance here than only the bold guideline itself: “In accordance with relevant national regulations, the permission of an immediate family member or other person with a close personal relationship with the individual must be sought.”...“in situations where a legally authorized representative is not available to allow for timely enrolment, researchers may obtain the permission of a representative who is socially accepted but not formally recognized before the law.” I think what the authors suggest is actually in line with CIOMS and a nice illustration of the exceptions to the general principle.
5. One minor issue: Please refer to the MacCat-CR already on page 3 where the 4 criteria are mentioned and the authors write “it is widely agreed that”, (not only on page 4).

Does the article adequately reference differing views and opinions?

Yes

Are all factual statements correct, and are statements and arguments made adequately supported by citations?

Partly

Is the Open Letter written in accessible language?

Yes

Where applicable, are recommendations and next steps explained clearly for others to follow?

Yes

Is the rationale for the Open Letter provided in sufficient detail?

Yes

Reviewer Expertise:

Bioethics, research ethics, global health ethics

I confirm that I have read this submission and believe that I have an appropriate level of expertise to confirm that it is of an acceptable scientific standard, however I have significant reservations, as outlined above.

## References

[1] : Ethical issues in the design and conduct of stepped-wedge cluster randomized trials in low-resource settings. *Trials* .2019;20(Suppl 2) : 10.1186/s13063-019-3842-1 703 10.1186/s13063-019-3842-1 31852547PMC6921381

# Response

**Published:** 2023-06-07

**Authors:** David Wendler

**Affiliations:** National Institutes of Health Clinical Center, USA

1. We agree and did not intend to suggest that the ethical issues considered here are unique to LMICs. Instead, as the reviewer notes, it is just as important to protect vulnerable subjects in LMICs as ones in high-income countries. The focus of our article is on the fact that this can be more challenging in LMICs. In particular, resource constraints can make it difficult to implement the measures needed to protect vulnerable participants. Our goal is to emphasize that measures can still be implemented to ensure appropriate protections. In particular, we argue that stakeholders in LMICs should: step back from the specific mandated procedures, identify the critical protections, and consider how they can be implemented in the setting in question. We have revised the discussion to try to make this point clear.

 2. With respect to case 1, we have revised the text to make clear that the center takes the following steps to try to minimize the risk of coercion: 1. Emphasize that patients always receive treatment according to the Chilean General Guarantee in Health Law; 2. Inform the family or other caregivers that the patient does not have to participate; 3. Do not offer any financial incentives to investigators for enrolling patients in research. We also note that the fact that 10-15% of patients decline to enroll suggests they do not feel that they must participate. For Case 2, in Ethiopia, when caregivers are involved in providing permission for persons who lack capacity to make a decision about participation, care is taken to ensure the caregiver (usually a close family member) lives with the individual and knows their preferences. The team also ensures that the person who lacks capacity does not object to the caregiver taking on this role. We have added these measures to the text.

 3. This is an important point. As the reviewer notes, commentators understand the relevant terms (e.g. competence, capacity) in different ways. However, we did not previously make that clear. We have thus revised the introduction to make this point explicitly and to specify how we are using the term. As we now explain, we are using the term ‘decisional capacity’ to refer to the collection of abilities that an individual needs to provide informed consent. This includes understanding and also the ability to make a voluntary decision. As the reviewer notes, the terminology differs from other approaches in the literature which use decisional capacity to refer more narrowly to the capacity to understand and reason.

 4. Thanks for this suggestion. We have revised the text in the discussion section accordingly.

 5. Done.

# Peer review: Reviewer response for version 1

**DOI:** 10.21956/wellcomeopenres.19962.r56609

**Published:** 2023-05-15

**Authors:** Jaranit Kaewkwungal

**Affiliations:** 1 Department of Tropical Hygiene, Faculty of Tropical Medicine, Mahidol University, Bangkok, Thailand

Review: Consent, decisional capacity and guardianship in mental health research

 This paper poses interesting points of view and practices regarding how researchers can enrol individuals with decisional incapacity in mental health research study. Case scenarios focused in issues experienced by researchers in LMICs (i.e., Latin America and Ethiopia).

 The authors proposed 7 safeguards for recruiting study participants with such characteristics: 1) Approval by the IRB/REC; 2) Appropriate risk–benefit profile; 3) Assessment of participants’ capacity to consent; 4) Justification for enrolling adults who cannot consent; 5) Permission of an appropriate proxy decision maker; 6) Consistency with participants’ preferences and values and 7) Respect for participant assent and dissent. I agree with such proposal and think that it should be applied as a universal standard.

 There are two comments that I would like the authors to elaborate as follow.

1. The authors mentioned that “To date, implementation of these protections has focused on high-income countries. For example, it is often assumed that there are trained clinicians available who can assess the decisional capacity of potential research participants.”
  Can the authors give an example of a study in high income country that actually practiced such safeguards in their study? How the researchers in that study managed the recruiting process and what tool that they used to assess decisional capacity? It will be a good lesson learn for researchers in LMICS and beyond.
2. According to the standard practices in most countries, following the Declaration of Helsinki and the CIOMS guidelines, researchers usually employ a “legally authorized representative of the person who is incapable of giving informed consent” approach.
  To the best of my knowledge, most legally authorized representatives (LARs) are commonly relatives or caretakers of the individuals. However, the authors discussed about the use of consent via “the given permission of the proxy decision maker who will be authorized by local or national legislation to enroll adults in research” and that “resource constraints in LMICs frequently make it difficult or impossible to follow these approaches. LMICs may not have enough trained clinicians to assess the decisional capacity of potential participants nor legislation governing who can serve as a research surrogate or proxy”. I am curious to learn about local/national legislation that permit trained clinicians rather than relatives to be LARs; and, what tool and how to train LARs.

Does the article adequately reference differing views and opinions?

Yes

Are all factual statements correct, and are statements and arguments made adequately supported by citations?

Yes

Is the Open Letter written in accessible language?

Yes

Where applicable, are recommendations and next steps explained clearly for others to follow?

Not applicable

Is the rationale for the Open Letter provided in sufficient detail?

Yes

Reviewer Expertise:

Research methodology and research ethics

I confirm that I have read this submission and believe that I have an appropriate level of expertise to confirm that it is of an acceptable scientific standard.

# Response

**Published:** 2023-06-07

**Authors:** David Wendler

**Affiliations:** National Institutes of Health Clinical Center, USA

1. Thanks for this suggestion. We have revised the text to briefly describe the process that is used at the US NIH Clinical Center and added a reference which describes the process in detail.

 2. In response to the previous comment, we have added a description and reference for the assessment process at the US NIH Clinical Center. In the absence of an LAR most jurisdictions with which we have experience rely on court appointed guardians or institutional ethics committees to make decisions. In Ethiopia, the ethics committee usually approves a close relative to be the surrogate.
